# 

**Published:** 1877-11

**Authors:** 


*CONTENTS.*

| | Page |
|---|---|
| Consumption | 295 |
| Health and Fun | 302 |
| A Lesson to Parents | 303 |
| Frost Work | 306 |
| Sun Baths | 306 |
| Jewel in a Blouse | 308 |
| Mr. Beecher on Hay Fever | 308 |
| Maccaroni | 313 |
| Inhalation and Consumption | 314 |
| Infants' Food | 316 |
| Celibacy | 316 |
| Carelessness | 318 |
| Reading | 319 |

